# Health Care Services Utilization in Iran

**Published:** 2017-06

**Authors:** Mehdi HAGHI, Ghasem RAJABI

**Affiliations:** 1. Dept. of Health Education, Faculty of Health, Shahid Sadoughi University of Medical Sciences, Yazd, Iran; 2. Modeling in Health Research Center, Institute for Futures Studies in Health, Kerman University of Medical Sciences, Kerman, Iran; 3. Dept. of Health Management and Economics, School of Public Health, Tehran University of Medical Sciences, Tehran, Iran

## Dear Editor-in-Chief

Access to public services is not equally distributed in Iran (1). This general rule also includes the access to health care services as the distribution of facilities is unequal skewed in terms of geographic location (2). The success of national development programs largely depends on success of health sector in achieving its objectives, and proper utilization of health care services in different parts of country is a prerequisite for this achievement.

The distribution of health facilities and services at a provincial level has been aptly studied by various researchers (3–8), but other surveys should also be carried out systemically and periodically at a national level to provide policy makers with sufficient information about the status of health system. The under or higher utilization of a region to health care services is governed by factors such as the out-of-pocket payments, geographic access, distribution of human resources, physical access, technological access, and etc. In the developing countries, availability of health care services is particularly influenced by drug shortages, unfair distribution of experienced personnel, lack of sophisticated medical equipment, lack of infrastructure, and weak monitoring and management of human resources (9). Therefore, in addition to level of utilization, the specific reasons behind good or poor utilization should also be determined.

This will allow the policymakers to develop and adopt long-term and short-term policies depending on the circumstances, especially with introduction of health transformation plan in Iran (10). National studies about utilization will show the distribution of available resources and the factors (political, technical, financial, etc.) causing the unequal distribution. Finally, without sufficient information and evidence, providing all strata of population with high quality of health care services will not be fulfilled.
